# Identification and Characterization of Circular RNAs in Mammary Tissue from Holstein Cows at Early Lactation and Non-Lactation

**DOI:** 10.3390/biom12030478

**Published:** 2022-03-21

**Authors:** Yan Liang, Qisong Gao, Haiyang Wang, Mengling Guo, Abdelaziz Adam Idriss Arbab, Mudasir Nazar, Mingxun Li, Zhangping Yang, Niel A. Karrow, Yongjiang Mao

**Affiliations:** 1 Key Laboratory for Animal Genetics, Breeding, Reproduction and Molecular Design of Jiangsu Province, Department of Animal Breeding and Production, College of Animal Science and Technology, Yangzhou University, Yangzhou 225009, China; mz120181016@yzu.edu.cn (Y.L.); 18305182715@163.com (Q.G.); hyangwang@163.com (H.W.); gl18852720440@163.com (M.G.); arbabtor@yahoo.com (A.A.I.A.); drmudasirnazar457@gmail.com (M.N.); limingxun@live.com (M.L.); yzp@yzu.edu.cn (Z.Y.); 2 Joint International Research Laboratory of Agriculture and Agri-Product Safety of Ministry of Education of China, Yangzhou University, Yangzhou 225009, China; 3 Biomedical Research Institute, Darfur University College, Nyala 63313, Sudan; 4 Center for Genetic Improvement of Livestock, Department of Animal Biosciences, University of Guelph, Guelph, ON N1G 2W1, Canada; nkarrow@uoguelph.ca

**Keywords:** circular RNA, mammary tissue, Holstein cows, RNA sequencing

## Abstract

In this study, circular RNAs (circRNAs) from Holstein cow mammary tissues were identified and compared between early lactation and non-lactation. After analysis, 10,684 circRNAs were identified, ranging from 48 to 99,406 bp, and the average size was 882 bp. The circRNAs were mainly distributed on chromosomes 1 to 11, and 89.89% of the circRNAs belonged to sense-overlapping circRNA. The exons contained with circRNAs ranged from 1 to 47 and were concentrated from 1 to 5. Compared with the non-lactating cows, 87 circRNAs were significantly differentially expressed in the peak lactation cows. There were 68 upregulated circRNAs and 19 downregulated circRNAs. Enrichment analysis of circRNAs showed that GO analysis mainly focused on immune response, triglyceride transport, T cell receptor signaling pathway, etc. Pathway analysis mainly focused on cytokine-cytokine receptor interaction, T helper 17 cell differentiation, fatty acid biosynthesis, the JAK-STAT signaling pathway, etc. Specific primers were designed for two proximal ends of the circRNA junction sites to allow for PCR validation of four randomly selected circRNAs and carry out circRNA-miRNA interaction research. This study revealed the expression profile and characteristics of circRNAs in mammary tissue from Holstein cows at early lactation and non-lactation, thus providing rich information for the study of circRNA functions and mechanisms, as well as potential candidate miRNA genes for studying lactation in Holstein cows.

## 1. Introduction

Circular RNAs (circRNAs) are a new class of endogenous non-coding RNA molecules formed by covalent bonds that were firstly identified in plant viroids [1]. Compared with traditional linear RNA, circRNAs do not have 5′ and 3′ ends because they are covalently bonded in a circular atresia structure (circRNA is a closed loop, connected at both ends by linear RNA), making them more resistant to RNase R and degradation [2]. Recently, studies have found that circRNAs are closely associated with the occurrence of certain human diseases, which has made circRNA a new trending area of RNA research [3]. CircRNAs are derived from trans-splicing of RNA precursors (pre-RNA) and can be divided into five categories according to their nucleotide source: EciRNA (exonic circular RNA, i.e., all exon-derived circRNA), IciRNA (intronic circular RNA, i.e., all derived from introns), EIciRNA (exon-intron circular RNA, i.e., sense-overlapping circular RNA derived from exons and introns), antisense circular RNA and intergenic circular RNA. CircRNAs from different sources can have different biological functions. Some can adsorb miRNAs, and therefore, regulate the expression of target genes by acting as miRNA sponges [4]. In addition, circRNAs can bind to transcriptional regulatory elements or interact with proteins to regulate gene transcription, and they play a role in m6A (N6-methyladenosine) modification to promote the effective initiation of protein translation [5,6].

Human medical research involving circRNA has mainly focused on cancer, nervous system diseases and immunity [7,8], while livestock circRNA research is still in its infancy. Liang et al. identified 5934 circRNAs from nine different Guizhou miniature pig tissues, including fat and heart and skeletal muscle at three developmental stages, and mapped the temporal and spatial expression profile of these circRNAs to construct the first miniature pig circRNA database [9]. Li et al. analyzed porcine transcriptome data to identify and characterize the function of circRNAs in the heart, liver, spleen, lung and kidney tissue [10]. In terms of lactation research, Zhang et al. and Wang et al. characterized differentially expressed circRNAs in Holstein cattle and sheep mammary tissues at different lactation stages, respectively [11,12].

The mammary gland is an important organ in Holstein cows as it is required for calf survival, passive immunity and early nutrition, and to produce the range of dairy products we consume. The hormones associated with lactation and the growth and apoptosis of mammary epithelial cells show temporal changes during different lactation stages [13], and genetic polymorphisms are known to affect the milk yield and composition [14]. Previous studies suggested that bovine mammary gland development may partly depend on specific microRNA (miRNA) expression patterns [15], and circRNA has been shown to absorb miRNA and thereby contribute to regulating the generation of unsaturated fatty acids by bovine mammary epithelial cells [16,17].

Given the potential of circRNA to indirectly regulate mammary tissue gene expression, it is necessary to identify and characterize circRNAs in mammary tissues at different lactation stages since this circRNA may be involved in the epigenetic and genetic regulation of mammary tissue functions.

This study used high-throughput RNA sequencing (RNA-seq) to study the expression profile of circRNA from Holstein cows during early lactation and non-lactation, and gene ontology (GO) enrichment analysis of the parental miRNA genes of the differentially expressed circRNA was performed. Four differentially expressed circRNAs were selected for authenticity verification by PCR and to investigate circRNA-miRNA interactions.

## 2. Materials and Methods

### 2.1. Statement of Animal Ethics

All experiments were performed in agreement with the care and use guidelines for experimental animals established by the Ministry of Science and Technology of the People’s Republic of China (approval number 2006-398). The mammary gland tissue sample collection process was in line with the welfare ethics of experimental animals, and a production license for experimental animals was obtained (SYDW-2019005). The experimentation was also approved by Yangzhou University, Yangzhou, China.

### 2.2. Animal Sample Collection

In this study, mammary gland tissues were sampled from three Holstein cows in early lactation (*n* = 3, 30 days postpartum) and non-lactation (*n* = 3, 315 days postpartum) on a large dairy farm in Jiangsu province. From the three cows sampled in the study, we collected mammary tissue samples in early lactation and again in non-lactation. All samples were from second-parity cows. Milk was completely extruded from the mammary gland of lactating cows free of mastitis before mammary gland tissue samples were collected. The biopsy sampling methodology is detailed by Li et al. [18]. Briefly, cow hair was shaved from the skin of the sample sites, then the skin was disinfected with ethanol (75%) and locally anesthetized with 1 mL of procaine administered subcutaneously. Next, a 1.5-cm incision was made at the sampling site, and the connective tissues were removed with sterile scissors and forceps to expose the parenchymal tissues. Then, mammary gland tissue biopsies (1–2 g) were harvested, washed with PBS buffer (Invitrogen, Carlsbad, CA, USA) and immediately frozen in liquid nitrogen until RNA was isolated. Finally, after the collection of mammary gland tissue samples, an 11-mm wound clip was used to clamp closed the skin incision, and povidone iodide cream was evenly applied to the skin incision.

### 2.3. RNA Preparation

For RNA extraction, the mammary tissue was treated with TRIzol reagent (Invitrogen, Carlsbad, CA, USA), and the total RNA was then extracted using the RNAprep Pure Tissue Kit (Tiangen, RNAprep Pure Tissue Kit, Beijing, China). Briefly, frozen mammary tissue (10–20 mg) was combined with 300 μL lytic solution RL and was thoroughly ground with a grinding pestle. Then, 590 μL RNase-free ddH2O and 10 μL Proteinase K were added to the homogenate and mixed at 56 °C for 10–20 min. After dissolving the total RNA with appropriate diethylpyrocarbonate (DEPC), the quantity and quality of RNA were measured using a spectrophotometer (NanoDrop^®^ ND-1000, Thermo Scientific, DE, Waltham, MA, USA). The quantity of total RNA was greater than 400 ng μL^−1^, and the 260/280 requirement was 1.9~2.0.

### 2.4. Identification of circRNAs in Mammary Tissue from Holstein Cows

The CircBase database included circRNA sequences of five species: human, mouse, nematode, cactus and coelacanth. If a species to be analyzed belongs to one of the above species, the circRNA identified is first studied using CIRI software (Gao, CircRNA Identifier, 2015) [19], and the identified results are compared with the above database to obtain the known circRNA and the newly identified circRNA. If the species does not belong to the above species, circRNA is predicted ab initio using CIRI. In our sequencing, Holstein cows did not belong to the database and thus needed to be predicted from scratch.

A sequencing library was constructed, and genomic sequence mapping and analysis were performed. Ribosomal RNA was removed from the mammary tissue RNA samples using a transcriptome isolation kit (Ribominus Bacteria 2.0, Thermo Fisher). The remaining RNA were paired-end sequenced using an Illumina HiSeq Xten (Illumina Inc., San Diego, CA, USA) from Shanghai Personal Biotechnology Company, Ltd. (Shanghai, China). After sequencing, the data were preprocessed as follows: (a) extreme reads of signal strength caused by sequencing instrument hardware were removed; (b) reads with low overall quality (Q = 20, base proportion less than 50%) were removed; (c) the proportion of read bases with an error rate less than 1% was removed; (d) reads with N-base ambiguity caused by insufficient sequencing fluorescence intensity were removed; (e) reads with a length of fewer than 20 bases and containing adaptor sequences were removed; (f) ribosomal RNA reads were removed. The clean reads obtained through the above preprocessing were used to identify the circRNAs using the website find_circ (https://github.com/marvin-jens/find_circ, accessed on 25 April 2021) [20]. The known circRNAs and newly predicted circRNAs were obtained using the CIRI software to predict the circular RNAs, and by comparing data with the circBase database (http://circrna.org/cgi-bin/singlerecord.cgi?id=mmu_circ_0001771, accessed on 26 April 2021) [19]. The chromosome distribution and length distribution of the identified circRNAs were analyzed according to the find_circ search Deseq website (http://bioconductor.org/biocLite.R, accessed on 28 April 2021), which was used to conduct standardized processing of the number of junction read counts of circRNA in each sample (base mean value was used to estimate the expression level). The difference multiple was also calculated, and NB (negative binomial distribution test) was used to test for a different significance of read numbers [21]. Finally, the differentially expressed circRNAs were screened according to the difference multiple and difference significance test results (Appendix A, Table A1).

### 2.5. PCR Validation of circRNAs

Specific primers were designed for both ends of the circRNA junction site for reverse transcriptase-polymerase chain reaction (RT-PCR) amplification, to verify the existence of circRNA_09759, circRNA_09048, circRNA_09761 and circRNA_03309 (Table 1). The RT products were checked for size and purity by 1% agarose gel electrophoresis (voltage 100 V, 30 min) and then sequenced by the Shanghai Sangon Company (Shanghai, China). Three software programs, SeqMan (Invitrogen, Carlsbad, CA, USA), SnapGene Viewer (Invitrogen, Carlsbad, CA, USA) and Vector NTI(Invitrogen, Carlsbad, CA, USA) were then used to analyze the sequences and find the junction sites in the circRNAs.

### 2.6. Target microRNA Predictions and Gene Ontology Enrichment Analysis

The miRNA-targets of each differentially expressed circRNA were predicted using the miRanda algorithm [22], and the interaction network of the circRNAs and their target miRNAs was analyzed using starBase and then drawn using Cytoscape [23]. The calculation method of ceRNA_score and *p*-value [24] is as follows:

(1) $$\mathrm{ceRNA}\_\mathrm{score}\;=\frac{\#\mathrm{MRE}\_\mathrm{for}\_\mathrm{share}\_\mathrm{miRNA}}{\#\mathrm{MRE}\_\mathrm{for}\_\mathrm{circRNA}\_\mathrm{miRNA}}$$

where circRNA represents the information of circRNA; ceRNA_score represents predicted ceRNA relationship scores; #shared_miRNA represents the number of co-miRNAs; miRNAs represent the names of co-miRNAs; *p*-value represents the *p*-value for ceRNA prediction.

The calculation formula of the *p*-value is as follows:

(2) $$p=\overset{\min\left(m_{p},m_{n}\right)}{\underset{i=m_{c}}{\sum }}\frac{\left(\begin{array}{c} m_{n}\\ i \end{array}\right)\left(\begin{array}{c} M_{T}-m_{n}\\ m_{p}-i \end{array}\right)}{\left(\begin{array}{c} M_{T}\\ m_{p} \end{array}\right)}$$

where *M_T_* represents the number of all miRNAs; *m_p_* represents the number of miRNAs that regulate this mRNA; *m_n_* represents the number of miRNAs that have regulatory effects on the circRNA; *m_c_* represents the number of common miRNAs.

Gene Ontology (GO) enrichment analysis was used to investigate the main functions of the parent genes of the differentially expressed circRNAs using the DAVID tool (https://david.ncifcrf.gov, accessed on 12 June 2021) [25,26].

## 3. Results

### 3.1. Identification and Sequence Characteristics of circRNAs in Mammary Tissue from Holstein Cows

**Figure 1:**
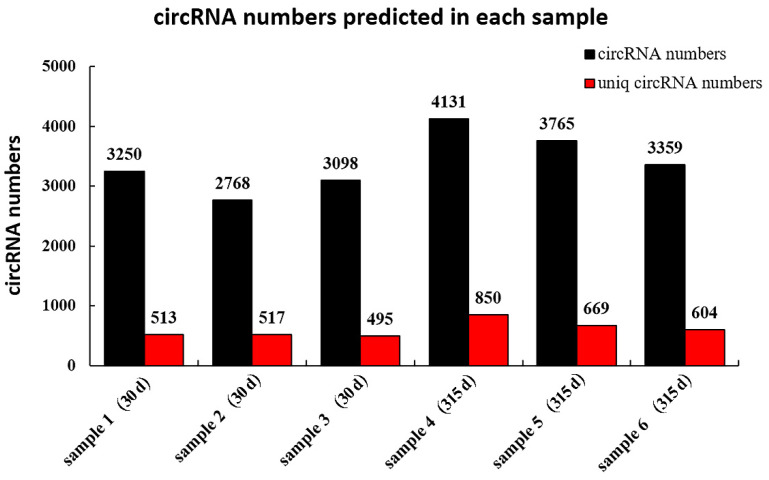
CircRNA numbers predicted in each sample. The vertical axis is the number of circRNAs; the horizontal axis shows the individual samples from cows on postpartum days 30 or 315; the numbers above each bar refer to the number of circRNAs predicted in each sample; the Uniq_circRNA_numbers refer to the number of circRNAs specifically predicted in each sample compared to other samples in the project.

**Figure 2:**
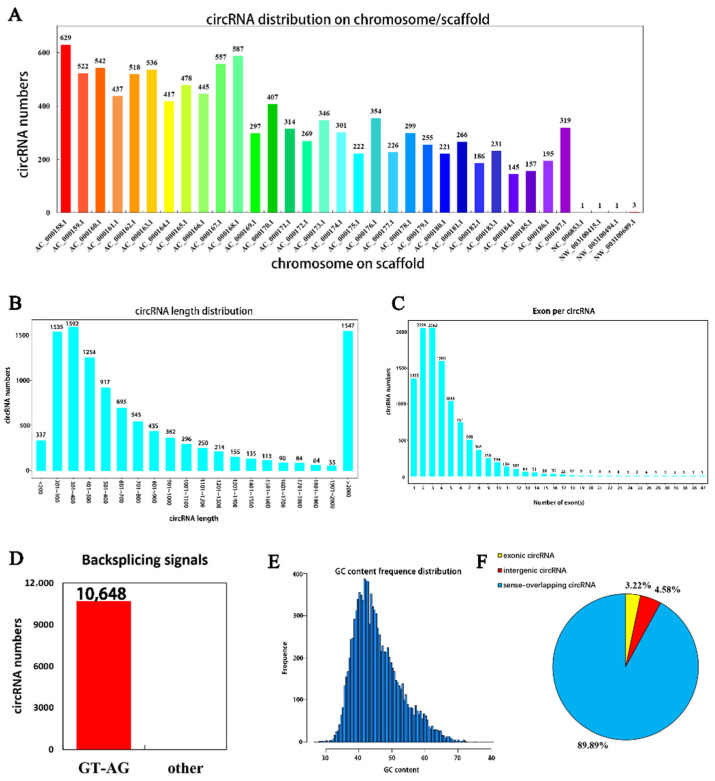
Identification, characterization and chromosomal distribution of circRNAs. (**A**) The number of circRNAs per chromosome; (**B**) the length distribution density of circRNAs); (**C**) the number of exons by circRNAs; (**D**) statistical diagram of circRNA shear signal, with the number of circRNAs on the vertical axis and the type of shear signal on the horizontal axis (specific data are presented in the Supplementary Materials, Table S1); (**E**) the vertical axis is the number of circRNAs and the horizontal axis shows the CG content of circRNAs; (**F**) percentage of different types of circRNAs.

A total of 10,684 circRNAs (Appendix B, Table A2) were identified from RNA in mammary tissue from Holstein cows by library construction, sequencing and bioinformatics analysis (detected in the actual sequencing were 10,684 GT-AG Splicing_signals). In total, 3250, 2768 and 3098 circRNAs were predicted in Holstein cows’ mammary tissue at 30 days, and 4143, 3765 and 3359 circRNAs were predicted at 315 days of lactation, respectively (Figure 1). The identified circRNAs were mainly distributed on chromosomes 1 to 11 (Figure 2A). Chromosome 1 contained the most circRNAs (*n* = 629). The sizes of circRNAs ranged from 48 to 99,406 bp, and the average size was 882 bp. The circRNA lengths mainly ranged by 201–400 bp, though some were greater than 2000 bp (Figure 2B). The number of exons contained with circular RNAs ranged from 1 to 47 and these were concentrated between 1 and 5 (Figure 2C). Variable shear signals’ AT reverse shear sites in circRNA sequences were counted, and all of them were CG-AT (Figure 2D). The CG content of circRNA was distributed in the range of 30–80%, mainly concentrated in the range of 40–50% (Figure 2E). It was found that 89.89% of circRNAs belong to EIciRNA, 4.58% belong to IciRN, and only 3.22% belong to the EciRNA (Figure 2F).

### 3.2. Differential Expression of circRNAs: Analysis in Mammary Tissue from Holstein Cows at Early Lactation and Non-Lactation

**Figure 3:**
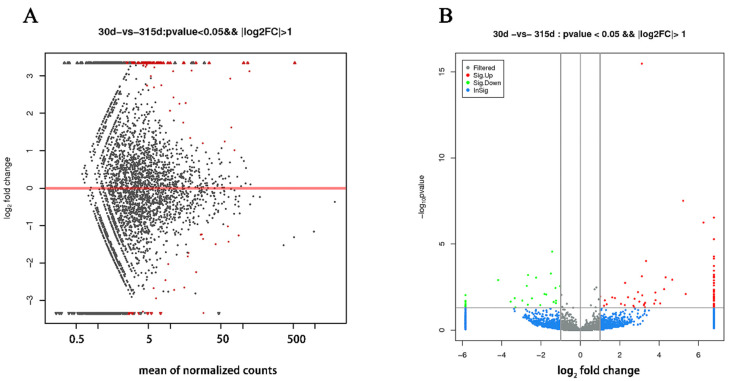
Differentially expressed circRNAs in mammary tissue from Holstein cows at early lactation and non-lactation. (**A**) The *X*-axis is the mean expression of all samples used for comparison after standardization. The *Y*-axis is the Log_2_ fold change. The red highlights are significantly differently expressed circRNAs. (**B**) Gray and blue circRNAs with non-significant differences; red and green circRNAs with upregulated and downregulated significant differences, respectively. The *X*-axis is the log_2_ fold change and the *Y*-axis is the log_10_ *p*-value.

**Figure 4:**
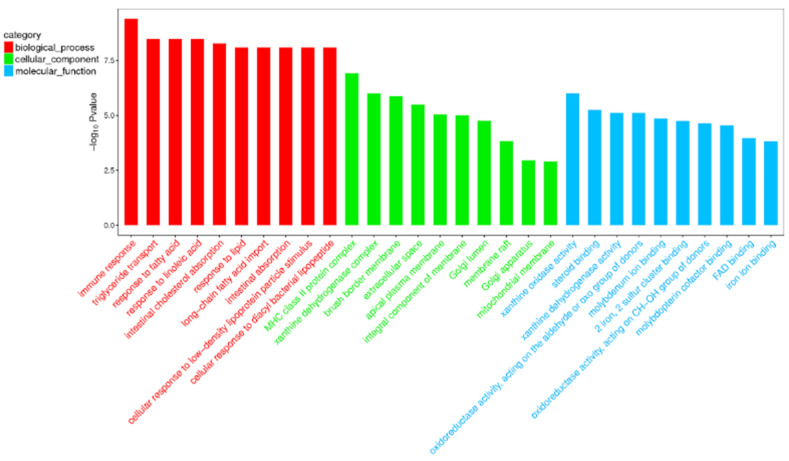
Top 30 categories of GO analysis. The basic information for each node, i.e., the GO ID and GO term, is displayed in the corresponding graph. The *X*-axis is the GO entry name and the *Y*-axis is the log_10_ *p*-value.

**Figure 5:**
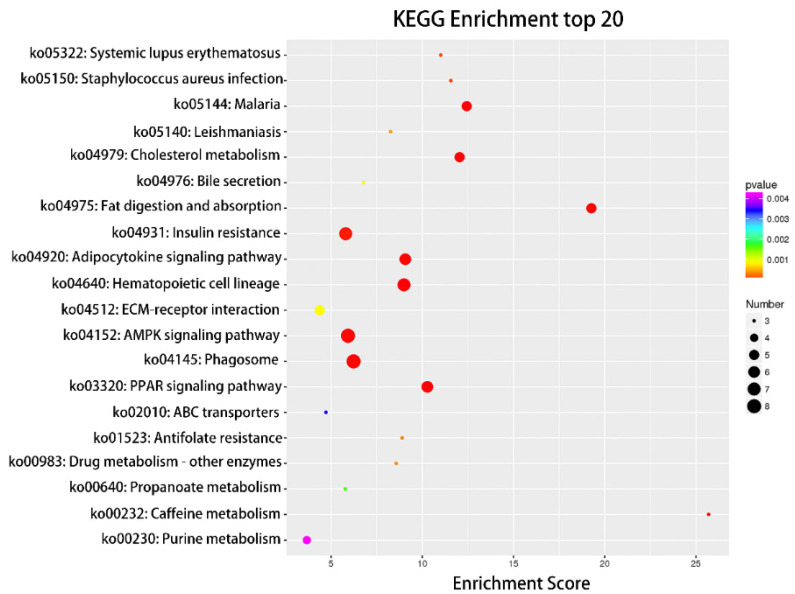
Top 20 categories of KEGG pathway analysis. KEGG enrichment top 20 bubble diagram, where the *X*-axis is the Enrichment Score. The larger the bubble, the more circRNAs the item contains, and the color of the bubble changes from purple to blue to green to red. The smaller the Enrichment *p*-value, the greater the significance.

Compared with the nonlactating Holsteins, 87 circRNAs with significantly different expressions were identified in the early lactation group, 68 of which were upregulated and 19 were downregulated (Table A1 and Figure 3, specific data are presented in the Supplementary Materials, Table S2). To describe the functions of differentially expressed circRNAs, GO enrichment analysis was carried out on their target genes. The GO analysis identified immune response, triglyceride transport and T cell receptor signaling functions (Figure 4). KEGG pathway analysis mainly focused on cytokine-cytokine receptor interactions, Th17 cell differentiation, fatty acid biosynthesis and the JAK-STAT signaling pathway (Figure 5).

### 3.3. CircRNA Authenticity Verification

**Figure 6:**
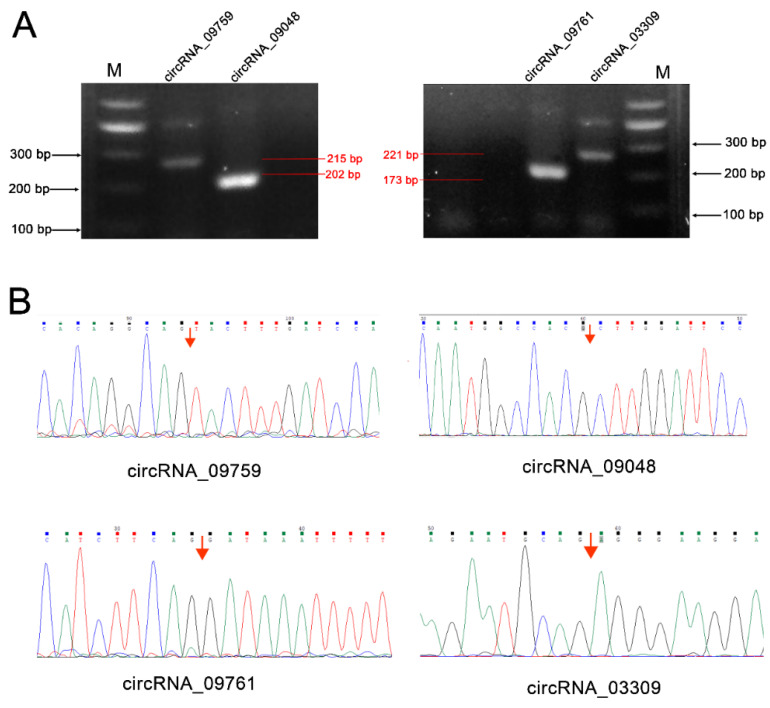
RT-PCR validation of the presence of circRNAs in cow mammary gland tissue. (**A**) Reverse transcriptase-polymerase chain reaction (RT-PCR) amplimers derived from the circular RNAs using divergent primers for cows’ mammary gland RNA (M: marker); (**B**) Head-to-tail splice junctions for the circRNAs were confirmed by DNA sequencing and are marked with a red arrow on the DNA sequence chromatograms.

CircRNA_09759, circRNA_09048, circRNA_09761 and circRNA_03309 were selected from the differentially expressed circRNAs for authenticity verification. These circRNAs are from mitochondrial glycerol-3-phosphate acyltransferase (GPAM), tumor necrosis factor receptor superfamily-member 21 (TNFRSF21) and solute carrier family 27 isoform A6 (SLC27A6), and may play a role in fatty acid transport, triglyceride synthesis, inflammation and immune regulation (Table S1). PCR amplification validated the existence of the circRNAs (Figure 6), and they produced the expected band sizes on the agarose gel (215 bp, 202 bp, 173 bp and 221 bp, respectively) (Figure 6A). DNA sequencing confirmed the presence of head-to-tail splice junctions as suggested by the RNA-seq analyses (Figure 6B) and the size of the circRNAs.

### 3.4. CircRNA-miRNA Interaction Research

**Figure 7:**
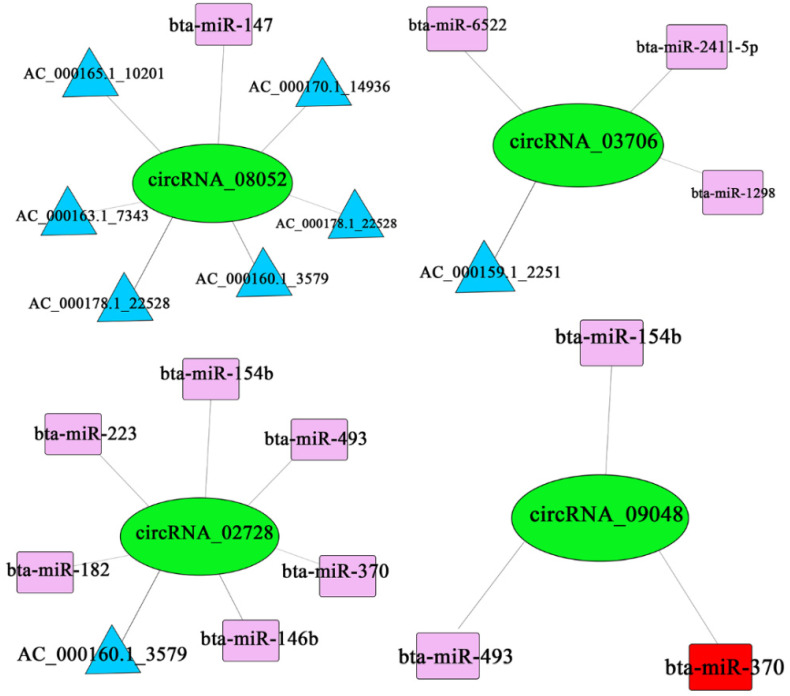
Four-network regulation of mRNA–circRNA–miRNA.

**Figure A1:**
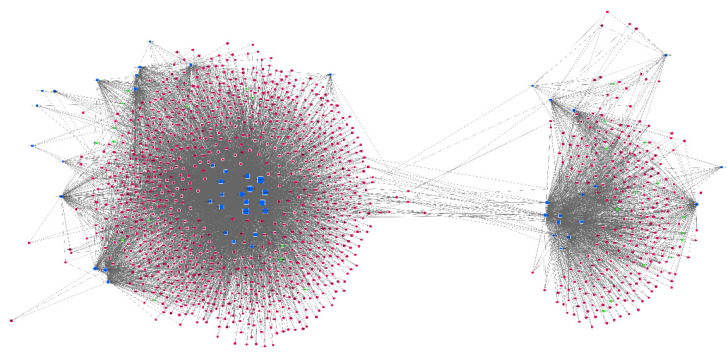
Network regulation of mRNA–circRNA–miRNA.

The predicted ceRNA relationships were paired and sorted from largest to the smallest according to the ceRNA_score and shared miRNA. Records with shared miRNAs less than 3 and *p*-values greater than 0.05 were filtered out [27]. The filtered results were shown in Figure A1 (Appendix C). We selected the four differentially expressed circRNAs from Table A1 (Appendix A) and the results showed that circRNA_08052 had regulatory relationships with AC_000178.1_22528, AC_000169.1_14270, bta-miR-154b and bta-miR-451; circRNA_03706 had regulatory relationships with bta-miR-6522, bta-miR-2411-5p, AC_000159.1_2251 and bta-miR-1298; circRNA_02728 had regulatory relationships with bta-miR-154b, bta-miR-182, AC_000160.1_3579, bta-miR-223, bta-miR-370, bta-miR-493 and bta-miR-146b; circRNA_09048 had regulatory relationships with bta-miR-493, bta-miR-370 and bta-miR-154b (Figure 7).

## 4. Discussion

Cows are not only a major source of dairy products but also an ideal large animal model to study the transcriptome and expression characteristics of the mammary gland. As a new non-coding RNA, circRNA has become a new research hotspot in recent years. Many studies have reported that circRNA is widely present in humans [28], mice [29], pigs [30], cattle [31], sheep [12] and other species [32]. 

Studies have identified circRNAs in sheep breast tissues during early lactation and non-lactation and found 3278 and 1756 circRNAs, 40 of which were upregulated and one of which was downregulated [12]. Hao [33] identified 4906 circRNAs in two sheep mammary gland tissues with different lactation performance levels, and 33 of these were differentially expressed between breeds. Another study showed that 6621 circRNAs were differentially expressed in the mammary tissue of Holstein cows at 90 and 250 days postpartum, of which 2231 were co-expressed [11]. In this study, high-throughput sequencing was used for the first time to explore the presence and expression of circRNAs in mammary tissue from Holstein cows at peak lactation and during the nonlactating period, and to screen and identify circRNAs that may play an important role in lactation. Through systematic identification and analysis of circRNAs, it was found that 3250 and 3359 circRNAs were predicted in the mammary tissue of Holstein cows at 30 and 315 days postpartum. These different results may be due to the relationship between mammalian mammary tissue development and the hormone level, genetic performance, parity, nutritional status and feeding management. Studies have shown that hormones in bovine mammary epithelial cells under different conditions have certain effects on the expression of genes related to milk composition synthesis [34]. For example, milk-derived hormones affect functional differentiation and local environmental signaling of mammary epithelial cells [35], thereby affecting mRNA expression. Therefore, different species, nutrient levels, physiological stages and external conditions may affect the expression of circRNAs in mammary tissue. 

Most of the circRNAs detected in the mammary tissue were very short, less than 1 kb in length, while some circRNAs were greater than 2 kb in length, which was also consistent with the results of analysis of circRNAs in the mammary glands of cows [11] and sheep [12], as well as bull testicles [36]. In addition, this study found that 89.89% of the identified circRNAs belonged to EIciRNA and were mostly distributed on chromosome 1. Wang et al. [12], meanwhile, identified six types of circRNA in sheep mammary tissue, among which EciRNA was the main one, with the circRNAs mainly concentrated on chromosomes 1 and 3. It is not surprising that bovine chromosome 1 produces the most circRNAs because it is the largest [37].

GO and KEGG enrichment analysis can illustrate the related functions of genes. In this study, the GO entries enriched by differential circRNAs were mainly involved in regulating various signaling pathways, metabolic processes and cell element composition, as well as signal transduction. The GO terms of circRNA parent genes have different enrichment levels at different stages of lactation [11]. The GO entry of immune response is the most significantly enriched biological process, which is important for fighting pathogen infections, for example, mastitis-causing pathogens including *Escherichia coli* and *Staphylococcus aureus* [38,39]. The next enriched biological processes were triglyceride transport and response to fatty acid (FA). In addition, the KEGG pathway analysis mainly revealed cytokine-cytokine receptor interactions, T helper cell 17 (Th17) differentiation-related pathways and biosynthesis of FA, and significantly enriched immune process pathways. Th17a T helper cells are derived from T helper cell 0 (Th0) when stimulated with IL6 and IL23, and are a potent source of inflammatory IL-17, playing an important role in autoimmunity in humans. 

Studies have shown that the CD molecule is often used as an important receptor or ligand for cells [40]. Both CD358 and CD4 are leukocyte differentiation antigens. CD358 is member 21 of the tumor necrosis factor receptor superfamily [41]. Overexpression of CD358 enables its cytoplasmic death domain to induce apoptosis and triggers the NF-κB pathways [42]. As an important member of the FA biosynthesis process, the main role of GPAM is to promote the production of triglyceride (TAG) in animals by catalyzing the biosynthesis of triacylglycerol and phospholipids. SLC27A6 has been characterized as a membrane-associated FA-binding protein that participates in FA transport across a cell membrane and is expressed primarily in heart muscle tissue [43]. Based on this, we selected circRNAs related to CD358, GPAM and SLC27A6 from the differentially expressed circRNAs for authenticity verification, and the existence of four circRNAs (circRNA_09759, circRNA_09048, circRNA_09761 and circRNA_03309) was successfully proven. 

In addition, it has been reported that circRNAs derived from four casein coding genes (CSN1S1, CSN1S2, CSN2 and CSN3) are highly expressed in the mammary tissue of cows after 90 days of lactation, and these circRNAs also have miR-2284 binding sites, which may be involved in the regulation of casein expression [11]. Liang et al. [9] identified 149 circRNAs related to the cation equilibrium, ATP hydrolyzation-coupled cation transport, tight cell junctions and calcium signaling pathways. Sun et al. [32] found that 40 circRNAs were involved in the miRNA-mediated ceRNA regulation network in Lantang pigs. Li et al. [33] found that cirCFGFR4 can absorb miR-107 and promote the expression of the Wnt3A gene, thereby promoting the differentiation of myoblasts and inducing apoptosis. Another study [44] also reported that circ11103 interacts with miR-128 to regulate milk fat metabolism in dairy cows. The above studies found that in different organisms and different physiological stages, circRNA has a specific expression-regulation mechanism and rich and important functions, not just appearing as a by-product of transcription. We also proved this in the circRNA-miRNA interaction research. Looking ahead, we will focus on circRNA_09048 for further study since we found that circRNA_09048 derives from the immune-related gene CD358 and has a regulatory relationship with miR-370 in circRNA-miRNA interaction research.

## 5. Conclusions

In this study, through high-throughput sequencing of circRNAs in Holstein cow mammary tissues at early lactation and non-lactation, 10,684 circRNAs were detected,. These were mainly distributed on chromosomes 1 to 10, with an average size of 882 bp and were mainly EIciRNA. Among the 87 differentially expressed circRNAs detected, enrichment analysis found that they were mainly concentrated on the immune response, T cell receptor signaling pathway, cytokine-cytokine receptor interaction and other pathways. This study revealed the expression profile and characteristics of circRNAs in the mammary tissue of Holstein cows during the early lactation and non-lactation periods and provided a wealth of information for studying the function and mechanism of circRNA.

## Figures and Tables

**Table 1:** PCR primers used to amplify specific circRNAs.

CircRNA	Forward (5′→3′)	Reverse (5′→3′)	Amplicon Size (bp)
circRNA_09759	TCTGGCTGAGCACATTCTCTTCAC	TCGCAGTAGTTCAACTATATGCCC	215
circRNA_09048	CAGAACCGGGAGAAGTGGATCTAC	ACGCAGGCTTATGTTGGTACAGTG	202
circRNA_09761	TTCAGGCAATAATCTCAACATCCC	TTATGGACATCTCGCTCTTGAATG	173
circRNA_03309	AACCAAACATGTCTTTAGACTTGG	TCCTTCAGTAGCTCCATAAAACTC	221

## Data Availability

Not applicable.

## Supplementary Materials

The following supporting information can be downloaded at: https://www.mdpi.com/article/10.3390/biom12030478/s1, Table S1: specific data of statistical diagram of circRNA shear signal, with the number of circRNAs on the vertical axis and the type of shear signal on the horizontal axis, Table S2: specific data of differentially expressed circRNAs in mammary tissue from Holstein cows at early lactation and non-lactation.

## Appendix A

**Table A1:** Differentially expressed circular RNAs in mammary tissue from Holstein cows at early lactation and non-lactation.

ID	Parent Gene Name	BaseMean Control ^2^	BaseMean Case ^3^	Fold Change	Log_2_ Fold Change	*p*-Value	Change ^4^
circRNA_00776	COBLL1	6.94	23.41	3.37	1.75	0.01	Up
circRNA_01190	FCGR2A	0.00	14.96	Inf ^1^	Inf	0.00	Up
circRNA_01200	YY1AP1	10.23	1.60	0.16	−2.67	0.03	Down
circRNA_01525	PRKAA2	3.50	18.76	5.37	2.42	0.01	Up
circRNA_01793	SEMA3A	0.00	5.33	Inf	Inf	0.04	Up
circRNA_01811	CD36	0.79	29.71	37.45	5.23	0.00	Up
circRNA_01812	CD36	0.00	4.99	Inf	Inf	0.03	Up
circRNA_01814	CD36	0.00	9.94	Inf	Inf	0.00	Up
circRNA_01815	CD36	0.79	11.16	14.06	3.81	0.01	Up
circRNA_01817	CD36	1.17	10.93	9.38	3.23	0.04	Up
circRNA_02322	RAB3IP	0.00	4.69	Inf	Inf	0.04	Up
circRNA_02331	CPSF6	83.78	41.17	0.49	−1.03	0.00	Down
circRNA_02392	GNPTAB	34.47	105.94	3.07	1.62	0.01	Up
circRNA_02393	GNPTAB	17.12	39.38	2.30	1.20	0.04	Up
circRNA_02481	CASC1	15.04	3.67	0.24	−2.04	0.04	Down
circRNA_02728	SLC39A8	0.87	17.63	20.18	4.34	0.00	Up
circRNA_02729	SLC39A8	1.17	29.65	25.45	4.67	0.00	Up
circRNA_02762	HERC6	1.20	9.60	7.98	3.00	0.02	Up
circRNA_02769	ABCG2	0.00	4.93	Inf	Inf	0.03	Up
circRNA_02773	ABCG2	0.00	13.49	Inf	Inf	0.00	Up
circRNA_02775	ABCG2	0.58	44.62	76.59	6.26	0.00	Up
circRNA_02819	SLC34A2	0.00	4.51	Inf	Inf	0.05	Up
circRNA_02963	SULT1B1	0.00	5.93	Inf	Inf	0.01	Up
circRNA_02974	CSN1S1	0.00	19.95	Inf	Inf	0.01	Up
circRNA_02976	CSN1S1	0.00	1049.13	Inf	Inf	0.02	Up
circRNA_02985	CSN1S1	0.00	13.00	Inf	Inf	0.02	Up
circRNA_03037	CSN1S2	0.00	234.34	Inf	Inf	0.00	Up
circRNA_03038	CSN1S2	0.00	8.71	Inf	Inf	0.00	Up
circRNA_03150	PCGF3	38.38	16.24	0.42	−1.24	0.02	Down
circRNA_03309	SLC27A6	0.00	7.11	Inf	Inf	0.01	Up
circRNA_03364	BTNL9	25.53	222.85	8.73	3.13	0.00	Up
circRNA_03433	NR3C1	0.00	8.21	Inf	Inf	0.05	Up
circRNA_03434	NR3C1	0.00	6.25	Inf	Inf	0.01	Up
circRNA_03706	BNC2	13.92	0.77	0.06	−4.18	0.00	Down
circRNA_03748	RIC1	0.00	6.07	Inf	Inf	0.03	Up
circRNA_03834	LOC516849	0.00	13.89	Inf	Inf	0.00	Up
circRNA_03919	RASEF	3.47	35.16	10.13	3.34	0.00	Up
circRNA_03938	SLC28A3	0.58	9.70	16.66	4.06	0.03	Up
circRNA_03940	NTRK2	11.26	1.46	0.13	−2.95	0.02	Down
circRNA_04110	RIMS1	52.37	5.17	0.10	−3.34	0.01	Down
circRNA_04412	UTRN	1.59	10.28	6.48	2.70	0.04	Up
circRNA_04598	PAPD4	27.19	7.68	0.28	−1.82	0.01	Down
circRNA_04711	LOC790312	1.93	13.03	6.75	2.75	0.02	Up
circRNA_04818	ICE2	0.00	5.79	Inf	Inf	0.02	Up
circRNA_05183	XDH	0.00	14.50	Inf	Inf	0.00	Up
circRNA_05186	XDH	4.64	22.17	4.78	2.26	0.04	Up
circRNA_05187	XDH	5.64	27.23	4.83	2.27	0.00	Up
circRNA_05251	HNRNPLL	4.03	35.63	8.84	3.14	0.01	Up
circRNA_05415	UGP2	10.65	26.92	2.53	1.34	0.03	Up
circRNA_05449	AAK1	0.00	4.78	Inf	Inf	0.04	Up
circRNA_05641	PAEP	1.85	204.80	110.63	6.79	0.00	Up
circRNA_05937	GGACT	0.00	8.66	Inf	Inf	0.01	Up
circRNA_06537	TRPS1	26.86	4.25	0.16	−2.66	0.00	Down
circRNA_06540	TRPS1	63.06	22.30	0.35	−1.50	0.00	Down
circRNA_06693	PDGFD	0.79	7.72	9.73	3.28	0.05	Up
circRNA_06862	FCHSD2	3.84	16.05	4.18	2.06	0.03	Up
circRNA_06890	MUC15	0.00	5.10	Inf	Inf	0.03	Up
circRNA_06905	HIPK3	51.31	103.42	2.02	1.01	0.01	Up
circRNA_07367	NR3C2	15.73	119.79	7.61	2.93	0.01	Up
circRNA_07504	TMEM120B	0.00	8.29	Inf	Inf	0.00	Up
circRNA_07505	TMEM120B	0.87	8.56	9.79	3.29	0.03	Up
circRNA_07624	ATP2C2	0.00	9.07	Inf	Inf	0.00	Up
circRNA_07671	CHD9	0.87	8.27	9.47	3.24	0.03	Up
circRNA_07739	MAG	40.95	15.95	0.39	−1.36	0.02	Down
circRNA_07878	ACACA	0.00	5.96	Inf	Inf	0.01	Up
circRNA_07881	ACACA	0.79	15.20	19.16	4.26	0.00	Up
circRNA_08052	TUBG2	1.24	8.28	6.67	2.74	0.05	Up
circRNA_08179	PANK3	0.79	10.71	13.50	3.76	0.03	Up
circRNA_08299	PARP8	8.95	0.77	0.09	−3.54	0.02	Down
circRNA_08529	NRG4	0.00	40.02	Inf	Inf	0.00	Up
circRNA_08530	NRG4	0.00	10.06	Inf	Inf	0.00	Up
circRNA_08655	CLMN	0.00	5.77	Inf	Inf	0.02	Up
circRNA_08923	LTF	19.74	2.99	0.15	−2.72	0.00	Down
circRNA_08986	BOLA-DMA	124.46	51.73	0.42	−1.27	0.00	Down
circRNA_09047	TNFRSF21	2.25	19.66	8.72	3.12	0.00	Up
circRNA_09048	TNFRSF21	33.79	80.02	2.37	1.24	0.02	Up
circRNA_09055	LOC100848815	0.00	9.03	Inf	Inf	0.01	Up
circRNA_09056	BOLA-DRB3	37.83	7.94	0.21	−2.25	0.00	Down
circRNA_09182	CTDP1	0.00	9.15	Inf	Inf	0.01	Up
circRNA_09759	GPAM	0.00	9.73	Inf	Inf	0.00	Up
circRNA_09761	GPAM	1.64	67.24	40.99	5.36	0.01	Up
circRNA_09774	PLEKHS1	7.58	0.77	0.10	−3.30	0.05	Down
circRNA_09991	PSD3	0.00	7.36	Inf	Inf	0.00	Up
circRNA_10245	NARS2	93.79	34.70	0.37	−1.43	0.00	Down
circRNA_10286	MYRF	14.54	2.64	0.18	−2.46	0.01	Down
circRNA_10359	ANO9	37.13	15.68	0.42	−1.24	0.03	Down
circRNA_10380	DDX26B	0.58	8.24	14.15	3.82	0.02	Up

^1^ Inf = infinity. ^2^ BaseMean is used to homogenize the gene expression in both the total sample and the single sample in the DEGSeq R package (Wanget et al., 2010). BaseMean Control is the BaseMean value of mammary tissue with peak lactation. ^3^ BaseMean Case is the BaseMean value of mammary tissue in the nonlactating period. ^4^ The difference in the expression of a specific circular RNA in the mammary tissue during the nonlactating period, compared with peak lactation.

## Appendix B

**Table A2:** The number of circRNAs between the two groups.

Group	Sample	circRNA Numbers	Unique circRNA Numbers	Pairwise Comparison in Each Group	Overlapped in Pairs in Each Group	Total Number of Unique circRNAs	Total Number Overlapping in Each Group	Total Number of circRNAs in Each Group
Early lactation	1	3250	513	sample 1 VS sample 2	2196	1525	3764	5289
	2	2768	517	sample 1 VS sample 3	2679			
	3	3098	495	sample 2 VS sample 3	2203			
				sample 1 VS sample 2 VS sample 3	1657			
Non-lactation	4	4131	850	sample 4 VS sample 5	2765	2123	4926	7049
	5	3765	669	sample 4 VS sample 6	3018			
	6	3559	604	sample 5 VS sample 6	2931			
				sample 4 VS sample 5 VS sample 6	1894			
Total number of circRNAs between the two groups.								12,338
Overlap between the two groups.								1654
Total number of circRNAs.								10,684

## References

[1] Sanger H.L., Klotz G., Riesner G., Gross H.J., Kleinschmidt A.K. (1976). Viroids are Single-Stranded Covalently Closed Circular RNA Molecules Existing as Highly Base-Paired Rod-Like Structures. Proc. Natl. Acad. Sci. USA.

[2] Koch L. (2017). RNA: Translated circular RNAs. Nat. Rev. Genet..

[3] Chen L.L., Yang L. (2015). Regulation of circRNA biogenesis. RNA Biol..

[4] Bai N., Peng E.M., Qiu X.S., Ning L., Zhang Z.J., Tao Y.M., Li X.Y., Wang Z.M. (2018). circFBLIM_1_ act as a ceRNA to promote hepatocellular cancer progression by sponging miR-346. J. Exp. Clin. Cancer Res..

[5] Huang A.Q., Zheng H.X., Wu Z.Y., Chen M.S., Huang Y.L. (2020). Circular RNA-protein interactions: Functions, mechanisms, and identification. Theranostics.

[6] Timoteo G.D., Dattilo D., Alvaro C.B., Colantoni A., Guarnacci M., Rossi F., Incarnato D., Oliviero S., Fatica A., Morlando M. (2020). Modulation of circRNA Metabolism by m6A Modification. Cell Rep..

[7] Liang C., Ge S. (2021). CircRNA in cancer: Fundamental mechanism and clinical potential. Cancer Lett..

[8] Li Q.W., Yang Y.L., Yi G.Q., Chen M.Y., Wang B.H., Fan X.H., Tang Z.L. (2021). CircAGAP1 promotes tumor progression by sponging miR-15-5p in clear cell renal cell carcinoma. J. Exp. Clin. Cancer Res..

[9] Liang G.M., Yang Y.L., Niu G.L., Tang Z.L., Li K. (2017). Genome-wide profiling of Sus scrofa circular RNAs across nine organs and three developmental stages. DNA Res..

[10] Li Q.W., Yang Y.L., Yi G.Q., Chen M.Y., Wang B.H., Fan X.H., Tang Z.L. (2020). Identification and characterization of porcine circRNAs. Acta Vet. Zustrinaria Sin..

[11] Zhang C.L., Wu H., Wang Y.H., Zhu S.Q., Liu J.Q., Zhang X.T., Hong C. (2016). Circular RNA of cattle casein genes are highly expressed in bovine mammary gland. J. Dairy Sci..

[12] Wang J.Q., Zhou H.T., Hickford J.G.H., Hao Z.Y., Gong H., Hu J., Liu X., Li S.B., Shen J.Y., Ke N. (2021). Identification and characterization of circular RNAs in mammary gland tissue from sheep at peak lactation and during the nonlactating period-ScienceDirec. J. Dairy Sci..

[13] Billa P.A., Faulconnier Y., Ye T., Chervet M., Provost F.L., Pires J., Lerou C. (2019). Deep RNA-Seq reveals miRNome differences in mammary tissue of lactating Holstein and Montbéliarde cows. BMC Genom..

[14] Wang M.Q., Ni W., Zhang H.M., Yang Z.P., Wang X.P., Jiang Y.S., Mao Y.J. (2017). Correlation Between the Mutation of SNPs in the Promoter Region of TLR1 and Mastitis Resistance and Milking Traits in Chinese Holstein (Bos taurus). J. Agric. Biotechnol..

[15] Chen Z., Chu S.F., Liang Y.S., Xu T.L., Sun Y.J., Li M.X., Zhang H.M., Wang X.L., Mao Y.J., Loor J.J. (2020). miR-497 regulates fatty acid synthesis via LATS2 in bovine mammary epithelial cells. Food Funct..

[16] Chen Z., Zhou J.P., Wang M.Q., Liu J.H., Zhang L.F., Loor J.J., Lian Y.S., Wu H., Yang Z.P. (2020). Circ09863 regulates unsaturated fatty acid metabolism by adsorbing miR-27a-3p in bovine mammary epithelial cells. J. Agric. Food Chem..

[17] Chen G., Li Y.J., Zhang A.L., Gao L.J. (2020). Circular RNA circ-BANP regulates ox-LDL-induced endothelial cell injury through targeting the miR-370/TXNIP axis. J. Cardiovasc. Pharmacol..

[18] Li C., Cai W., Zhou C., Yin H., Zhang Z., Loor J.J., Sun D.X., Zhang Q., Liu J., Zhang S. (2016). OPEN RNA-Seq reveals 10 novel promising candidate genes affecting milk protein. Sci. Rep..

[19] Gao Y., Wang J.F., Zhao F.Q. (2015). CIRI: An efficient and unbiased algorithm for de novo circular RNA identification. Genome Biol..

[20] Memczak S., Jens M., Elefsinioti A., Torti F., Krueger J., Rybak A., Maier L., Mackowiak S.D., Gregersen L.H., Munschauer M. (2013). Circular RNAs are a large class of animal RNAs with regulatory potency. Nature.

[21] Anders S., Huber W. (2013). Differential expression of RNA-Seq data at the gene level–the DESeq package. Heidelb. Ger. Eur. Mol. Biol. Lab. EMBL.

[22] Miranda K.C., Huynh T., Tay Y., Ang Y.S., Tam W.L., Thomson A.M., Lim B., Rigoutsos I. (2006). A pattern-based method for the identification of MicroRNA binding sites and their corresponding heteroduplexes. Cell.

[23] Smoot M.E., Ono K., Ruscheinski J., Wang P.L., Ideker T. (2011). Cytoscape 2.8: New features for data integration and network visualization. Bioinformatics.

[24] Shaoli D., Suman G., Rituparno S., Jayprokas C., Sandro B. (2014). lnCeDB: Database of Human Long Noncoding RNA Acting as Competing Endogenous RNA. PLoS ONE.

[25] Minoru K., Michihiro A., Susumu G., Masahiro H., Mika H., Masumi I., Toshiaki K., Shuichi K., Shujiro O., Toshiaki T. (2008). KEGG for linking genomes to life and the environment. Nucleic Acids Res..

[26] Sun J., Wang S., Li C., Ren Y., Wang J. (2014). Novel expression profiles of microRNAs suggest that specific miRNAs regulate gene expression for the sexual maturation of female Schistosoma japonicum after pairing. Parasites Vectors.

[27] Tay Y., Kats L., Salmena L., Weiss D., Tan S.M., Ala U., Karreth F., Poliseno L., Provero P., Cunto F.D. (2011). Coding-independent regulation of the tumor suppressor PTEN by competing endogenous mRNAs. Cell.

[28] Yang X., Ye T., Liu H., Lv P., Duan C., Wu X., Jiang K., Lu H., Xia D., Peng E. (2021). Expression profiles, biological functions and clinical significance of circRNAs in bladder cancer. Mol. Cancer.

[29] Fan X.Y., Zhang X.N., Wu X.L., Guo H.H., Hu Y.Q., Tang F.C., Huang Y.Y. (2015). Single-cell RNA-seq transcriptome analysis of linear and circular RNAs in mouse preimplantation embryos. Genome Biol..

[30] Sun J., Xie M., Huang Z., Li H., Chen T., Sun R., Wang J., Xi Q.Y., Wu T., Zhang Y. (2017). Integrated analysis of non-coding RNA and mRNA expression profiles of 2 pig breeds differing in muscle traits. J. Anim. Sci..

[31] Li H., Wei X.F., Yang J.M., Dong D., Hao D., Huang Y.Z., Lan X.Y., Plath M., Lei C.Z., Ma Y. (2018). CircFGFR4 promotes differentiation of myoblasts via binding miR-107 to relieve its inhibition of Wnt3a. Mol. Ther.-Nucleic Acids.

[32] Zhang Y., Wang L., Qiu L., Pan R., Bai H., Jiang Y., Wang Z., Bi Y., Chen G., Chang G. (2019). Expression patterns of novel circular RNAs in chicken cells after avian leukosis virus subgroup J infection. Gene.

[33] Hao Z.Y., Zhou H.T., Hickford J.G.H., Gong H., Wang J.Q., Hu J., Liu X., Li S.B., Zhao M.L., Luo Y.H. (2020). Identification and characterization of circular RNA in lactating mammary glands from two breeds of sheep with different milk production profiles using RNA-Seq. Genomics.

[34] Xing Y.Y., Li D.B., Sun M., Zhang H., Hou X.Z., Gao M. (2020). Effect of hormones on genes related to hormone receptors and milk component synthesis in bovine mammary epithelial cells grown in two and three dimensional culture system. Ital. J. Anim. Sci..

[35] Yonekura S., Sakamoto K., Komatsu T., Hagino A., Katoh K., Obara Y. (2006). Growth hormone and lactogenic hormones can reduce the leptin mrna expression in bovine mammary epithelial cells. Domest. Anim. Endocrinol..

[36] Yuan G., Wu M.L., Fan Y.Z., Li S.P., Li S.P., Lai Z.Y., Huang Y.Z., Lan X.Y., Lei C.Z., Chen H. (2018). Identification and characterization of circular RNAs in Qinchuan cattle testis. R. Soc. Open Sci..

[37] Yu R.L., Xin C.Y. (1991). Studies on chromosome of Luxi cattle. Cattle J..

[38] Korkmaz F., Elsasser T., Kerr D. (2018). Variation in fibroblast expression of toll-like receptor 4 and lipopolysaccharide-induced cytokine production between animals predicts control of bacterial growth but not severity of Escherichia coli mastitis. J. Dairy Sci..

[39] Lakshmi R., Jayavardhanan K., Thanislass J. (2017). Toll like receptor-4 gene expression assay in mastitis caused by Stapylococcus aureus in crossbred cattle. Int. J. Consum. Stud..

[40] Freage L.D., Jamal D., Williams N.B., Mallikaratchy P.R. (2020). A Homodimeric Aptamer Variant Generated from Ligand-Guided Selection Activates the T Cell Receptor Cluster of Differentiation 3 Complex. Mol. Ther.-Nucleic Acids.

[41] Tute R.M.D. (2011). Flow cytometry and its use in the diagnosis and management of mature lymphoid malignancies. Histopathology.

[42] Renata R.O., Messores C., Mariangeles A., Chiappin C.C., Mattos S.P.I., Gil L.B., Jéssica P.S., Moraes A.C.R., Cristina D.G., Marin P.M. (2018). Determination of normal expression patterns of CD86, CD210a, CD261, CD262, CD264, CD358, and CD361 in peripheral blood and bone marrow cells by flow cytometry. Immunol. Lett..

[43] Nafikov R.A., Schoonmaker J.P., Korn K.T., Noack K., Garrick D.J., Koehler K.J., Minick-Bormann J., Reecy J.M., Spurlock D.E., Beitz D.C. (2013). Association of polymorphisms in solute carrier family 27, isoform A6 (SLC27A6) and fatty acid-binding protein-3 and fatty acid-binding protein-4 (FABP3 and FABP4) with fatty acid composition of bovine milk. J. Dairy Sci..

[44] Chen Z., Lu Q.Y., Liang Y.S., Cui X.S., Wang X.L., Mao Y.J., Yang Z.P. (2021). Circ11103 interacts with miR-128 to regulate milk fat metabolism in dairy cows. J. Agric. Food Chem..

