# Supplementary figures and images for: Identification and Characterization of Circular RNAs in Mammary Tissue from Holstein Cows at Early Lactation and Non-Lactation
Source: Biomolecules. 2022 Mar 21;12(3):478. doi: 10.3390/biom12030478 (PMC8946036; doi:10.3390/biom12030478)

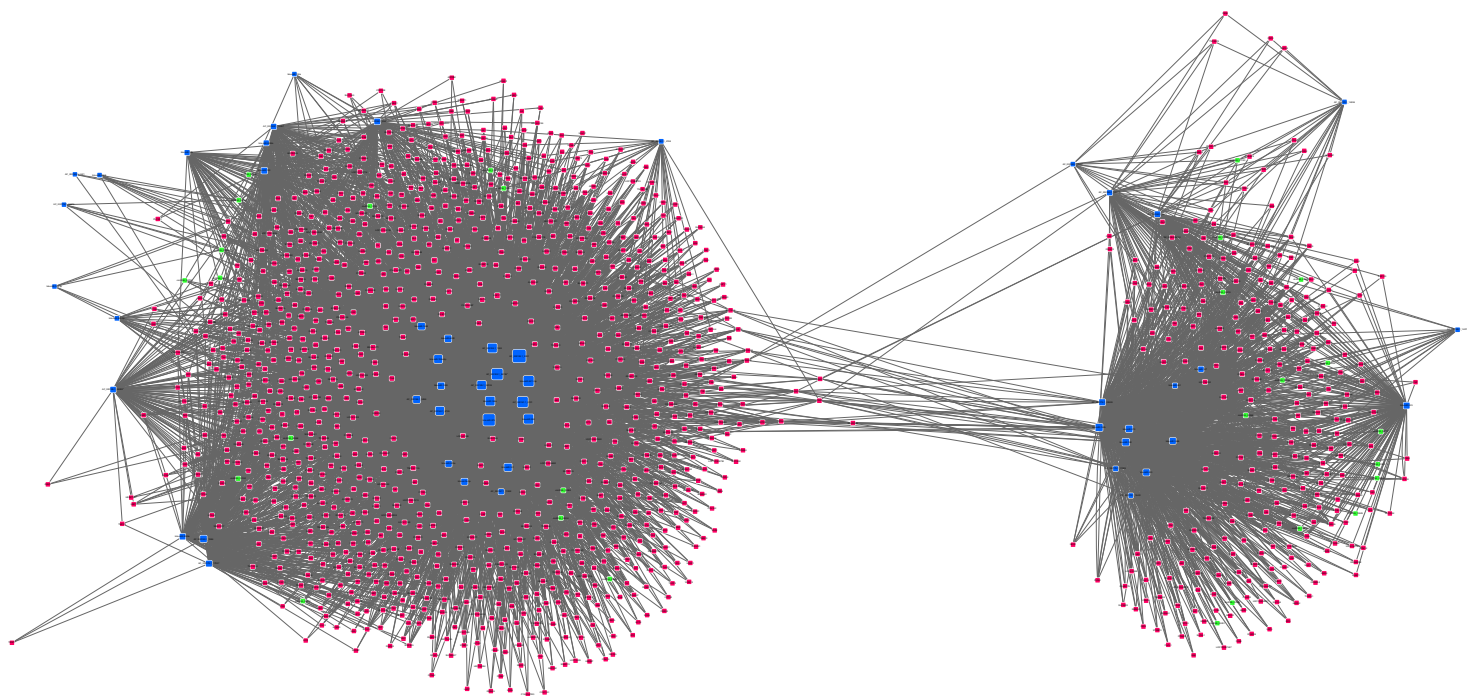

Supplement: Supplementary file 1 [file biomolecules-12-00478-s001.zip › Figure A1.pdf]
